# Significant Mean and Extreme Climate Sensitivity of Norway Spruce and Silver Fir at Mid-Elevation Mesic Sites in the Alps

**DOI:** 10.1371/journal.pone.0050755

**Published:** 2012-11-29

**Authors:** Marco Carrer, Renzo Motta, Paola Nola

**Affiliations:** 1 Università degli Studi di Padova – Dip. TeSAF – Forest Ecology Research Unit – Agripolis, Legnaro, Italy; 2 Università degli Studi di Torino – Dip. Scienze Agrarie, Forestali e Alimentari, Grugliasco, Italy; 3 Università degli Studi di Pavia – Dip. Scienze della Terra e dell'Ambiente, Pavia, Italy; University of Illinois, United States of America

## Abstract

Climate forcing is the major abiotic driver for forest ecosystem functioning and thus significantly affects the role of forests within the global carbon cycle and related ecosystem services. Annual radial increments of trees are probably the most valuable source of information to link tree growth and climate at long-term time scales, and have been used in a wide variety of investigations worldwide. However, especially in mountainous areas, tree-ring studies have focused on extreme environments where the climate sensitivity is perhaps greatest but are necessarily a biased representation of the forests within a region. We used tree-ring analyses to study two of the most important tree species growing in the Alps: Norway spruce (*Picea abies*) and silver fir (*Abies alba*). We developed tree-ring chronologies from 13 mesic mid-elevation sites (203 trees) and then compared them to monthly temperature and precipitation data for the period 1846–1995. Correlation functions, principal component analysis and fuzzy C-means clustering were applied to 1) assess the climate/growth relationships and their stationarity and consistency over time, and 2) extract common modes of variability in the species responses to mean and extreme climate variability. Our results highlight a clear, time-stable, and species-specific response to mean climate conditions. However, during the previous-year's growing season, which shows the strongest correlations, the primary difference between species is in their response to extreme events, not mean conditions. Mesic sites at mid-altitude are commonly underrepresented in tree-ring research; we showed that strong climatic controls of growth may exist even in those areas. Extreme climatic events may play a key role in defining the species-specific responses on climatic sensitivity and, with a global change perspective, specific divergent responses are likely to occur even where current conditions are less limited.

## Introduction

The growth and distribution of forests and the related roles of forests within the terrestrial carbon cycle are closely intertwined with climate forcing and variability at both short and long time-scales [1]. Yet this relationship between climate and forests is not homogeneous across geographical areas or among species. Indeed, significant differences, at both physical and biological levels, have been found across continents [2], [3], regions [4], [5], ecosystems, taxa and seasons [6]. This variability is clearly associated with spatial changes in environmental factors, but also associated with the corresponding positive or negative plant-plant interactions that are able to significantly shape the composition and dynamics of forest communities [7], [8].

Many studies have shown that some regions and species are more sensitive to climate variations than others [9]. For example, the altitudinal and latitudinal treeline is one key research area, where there is i) high sensitivity to environmental changes, ii) frequent presence of long-lived trees and iii) decreasing importance of competition - in terms of its effect on adult growth - with increasing limiting conditions [8], [10]. This usually permits the effective isolation, at various time scales, of the role of the most stressful growth limiting factor (temperature) on plant growth processes [10], [11]. Similarly, for xeric habitats, water is the primary limiting factor, and tree growth is more sensitive to corresponding changes in hydrological cycle [12].

Annual radial growth increments of trees are probably the most valuable source of environmental and ecological information for long time periods. These have been used in a wide variety of studies largely conducted in extra-tropical regions from typical climate reconstructions [13] to changes in species’ and ecosystems’ climate sensitivity according to internal [14], [15], external [16], [17], or geographical factors [18], [19].

All of these investigations have added valuable insight into species-specific climate/tree growth relationships and ecosystem responses to climate variability over local to continental spatial scales. However, most of the studies were carried out in marginal areas where the expression of the limiting factors for tree species probably reach the maximum, but at the cost of no longer being representative of the broader forested region. In other words, the role of forests within the global carbon cycle as well as the goods and services they provide is usually reduced in many extreme environments, whereas this role is maximized where conditions are less limiting such as, far from the treeline and at mid-latitude or mid-elevation. Nonetheless, even at lower latitudes or lower elevations, climate is still one of the most influential forcing factors for tree growth and ecosystem functioning. This calls for a better understanding of species and forest ecosystem behaviour in more mesic and milder environments. Indeed, knowing long- and short-term species responses to climate variability, where climate is not regularly limiting, is becoming more important for understanding the role of forests under various future change scenarios [3], [20].

The Alps are one of the most studied areas worldwide and this mountain range now has one of the best networks of high density and high-quality tree-ring [17], [21] and long-term meteorological records [22]. Nonetheless, tree-ring research in the Alps has been biased towards extreme habitats with an overrepresentation of high-elevation or xeric sites. Here, we investigated whether trees growing on mesic sites at lower elevations exhibited distinct climate/growth relationships. Indeed, traditional models in tree ecology and ecophysiology generally propose a convergent tendency on the species' growth responses to climate on more stressful sites [10]. Our underlying hypothesis is that significant and divergent species-specific growth responses occur also where conditions are less limiting. We used two of the most representative species of the montane belt of the Alps: Norway spruce (*Picea abies* (L.) Karst.) and silver fir (*Abies alba* Mill.). By using a dense network of tree-ring sites within a typical inner Alpine valley, we tryed to test the species sensitivity to mean climate variability and extreme climatic events, not at the limits of the species’ distributions but well within the temperature and precipitation ranges where most spruce and fir forests find their optimal growth conditions.

We adopted both classical and novel methodological approaches to explore the influences of mean and extreme climate on tree growth. We first computed the climate-growth relationships using correlation functions and then analyzed the common climate response patterns with principal component analysis and fuzzy C-means clustering.

## Materials and Methods

### Ethics Statement

All the field studies and sampling were carried out after permissions had been obtained from the Valle D'Aosta regional forests administration and the Gran Paradiso National Park, Italy.

### Setting

Aosta Valley is located in the western Italian Alps. Due to the inner setting within the Alps, the surrounding high peaks and its east-west orientation, the climate is continental and among the driest in the Alps. The mean annual precipitation and temperature are 561 mm and 10.1°C, respectively (long-term mean 1841–2007 for temperature and 1921–2010 for precipitation at Aosta, 544 m a.s.l., in the central valley) [22]. However, temperature and precipitation distributions are closely related to elevation.

In this region silver fir represents only 3% of the regional tree biomass. It grows mainly on north-facing slopes of the montane belt where it often forms pure stands but also occurs mixed with spruce and European larch (*Larix decidua* Mill.). Norway spruce is one of the most important species, representing 32% of the regional forest biomass and occurs in the montane and subalpine belts growing in pure and mixed forests, mainly with larch at higher elevation. In the western Italian Alps, silver fir is currently under-represented compared to its potential distribution. Indeed, many of the regional forests of the montane belt could potentially be mixed with the occurrence of both fir and spruce. Past human land-use has systematically selected for a few, preferred species causing the progressive disappearance of others, mainly silver fir in most of the sites. Alternatively, where human influence has not been historically significant in the last couple of centuries, forests are denser. This permits the presence of pure, or near-pure, stands dominated by the shade-tolerant silver fir, which overtakes more shade-intolerant species [23].

**Figure 1:**
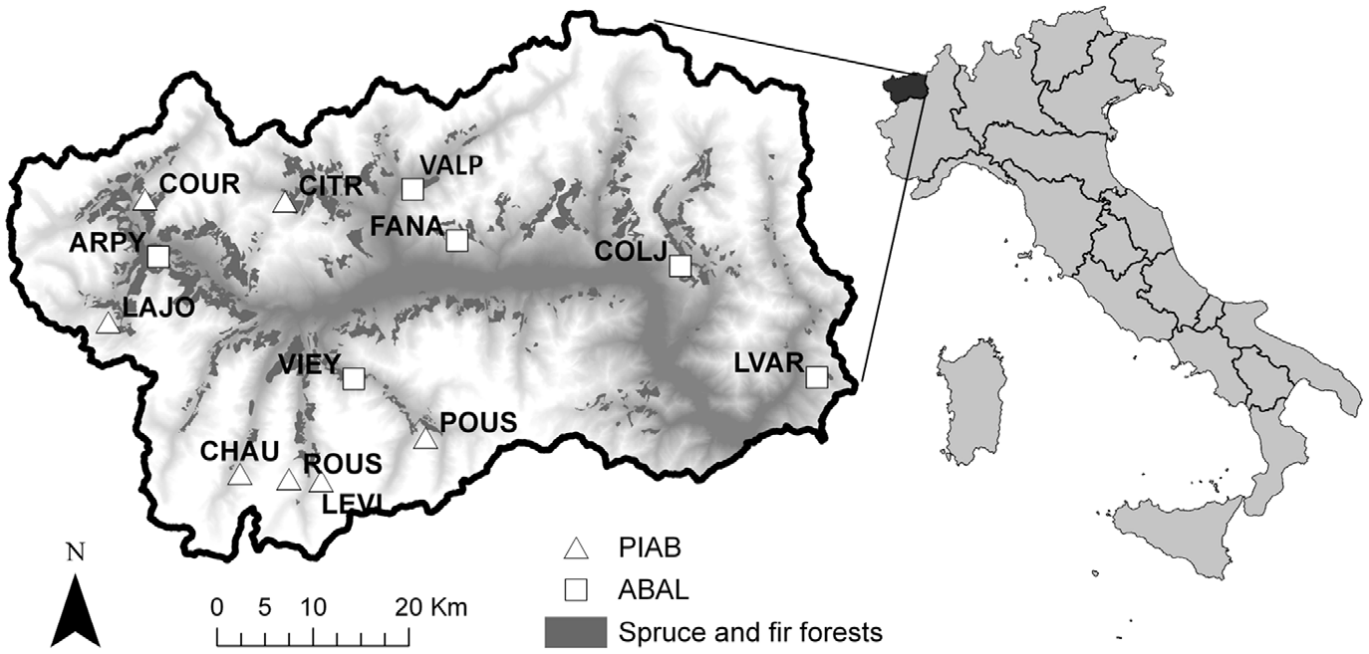
Location of the study area. Geographical location of the study area and site distribution in the Aosta Valley, Italy.

Seven Norway spruce and six silver fir sites were selected (Fig.1). They were located from 45.82° to 45.54° N latitude, and from 6.93° to 7.89°E longitude, at an altitude between 1200 and 1900 m a.s.l. (Table 1). Sampled forests were naturally regenerated, uneven-aged stands, with the presence of old trees and limited recent natural or human disturbances.

**Table 1:** Site location and descriptive statistics of the 13 tree-ring chronologies.

Code	Species	Lat	Long	Altitude(m)	Firstyear	Lastyear	Series length(years)					NC	NT	AC	MS	PC1	rbar	EPS
							Max		Mean		Min							
POUS	PIAB	45.59	7.37	1900	1638	1995	358	186		96		26	13	0.73	0.17	45	0.38	0.85
LEVI	PIAB	45.54	7.23	1900	1672	1995	324	199		87		30	15	0.80	0.15	53	0.49	0.93
ROUS	PIAB	45.55	7.19	1900	1636	1995	360	238		145		30	15	0.75	0.15	48	0.44	0.92
CHAU	PIAB	45.55	7.12	1900	1742	1995	254	207		111		30	15	0.83	0.15	55	0.51	0.93
LAJO	PIAB	45.69	6.94	1750	1771	1997	227	150		76		32	15	0.81	0.14	44	0.33	0.78
COUR	PIAB	45.80	6.98	1600	1703	1997	295	233		151		49	21	0.75	0.14	43	0.39	0.93
CITR	PIAB	45.81	7.17	1800	1763	1998	236	204		155		36	16	0.74	0.14	44	0.40	0.91
ARPY	ABAL	45.75	7.00	1700	1754	1998	245	191		141		43	19	0.85	0.14	58	0.56	0.96
COLJ	ABAL	45.64	7.27	1500	1776	1998	223	161		114		38	15	0.82	0.16	59	0.48	0.82
FANA	ABAL	45.82	7.34	1200	1755	1998	244	156		100		31	14	0.85	0.17	53	0.47	0.89
LVAR	ABAL	45.77	7.40	1700	1804	1997	194	150		95		28	14	0.85	0.16	54	0.44	0.83
VALP	ABAL	45.75	7.70	1500	1800	1998	199	158		92		39	17	0.84	0.20	48	0.42	0.90
VIEY	ABAL	45.65	7.89	1700	1655	1998	344	236		112		37	16	0.75	0.20	60	0.57	0.95

Note: PIAB and ABAL are the species codes for *Picea abies* and *Abies alba*. NC and NT are cores and trees numbers, respectively. Chronology statistics include first-order serial autocorrelation (AC), mean sensitivity (MS), the variance explained by the first principal component (PC1), mean interseries correlation (rbar) and expressed population signal (Eps). All except AC are computed on the indexed tree-ring series and on the 1846–1995 common period. See Fig. 1 for site locations.

### Tree-ring Data

A minimum of two cores per tree were collected at breast height on the cross-slope sides of the trunk from at least 13 trees at each site. We followed the classical dendroecological protocol, selecting only healthy dominant or co-dominant trees with no visible scars or signs of recent injuries in an attempt to enhance the climatic information retained in the tree-ring sequences and reduce to a minimum the possible effects of external influences such as competition, crown suppression or small-scale disturbances. Samples were prepared following standard procedures outlined in Stokes and Smiley [24]. Tree-ring width was then measured to 10-µm resolution and finally assigned to calendar years. Each ring-width series was first visually and then statistically checked for crossdating and measurement errors using the program COFECHA [25]. Finally, a total of 449 tree-ring series from 190 trees were considered for growth/climate response analysis. Tree-ring site chronologies were obtained from the crossdated ring-width series using the program ARSTAN [26] that was specifically developed for the removal of biologically induced age-trends [10] and to process the disturbance pulses often present in tree-ring series from closed-canopy forests [27]. Individual series were first standardized by fitting a negative exponential curve to measured data series and dividing observed by expected values. To emphasize high-frequency variability these dimensionless indices were then submitted to a second standardization procedure fitting a cubic smoothing spline with 50% frequency cut-off at 20 years and again computing the observed *vs.* expected ratio. Various statistical parameters were calculated to compare the tree-ring chronologies: i) mean sensitivity (MS), a measure of the relative difference in ring widths between consecutive years, adopted to assess the high-frequency variability of the series, ii) the first order serial autocorrelation (AC), a measure of the influence of previous year’s conditions on ring formation (Fritts, 1976), iii) the variance explained by the first principal component (PC1), and iv) the mean correlation between trees (rbar) and the “expressed population signal” (EPS) to estimate the level of year-by-year growth variations shared by trees in the same site. Higher values of PC1 and rbar indicate higher synchronization in the annual growth patterns among sampled trees and better common signal strength by the mean growth chronologies [10], while EPS is commonly adopted as a criterion for assessing a mean chronology's reliability [28].

### Climate Data

The HISTALP gridded dataset of monthly temperature and precipitation series [22] was used as predictor variables for growth/climate analyses over the 1846–1995 period. This dataset is based on precipitation and temperature data from hundreds of weather stations throughout the Greater Alpine Region, which were subjected to homogeneity tests and relative adjustments, and then gridded on a 1°×1° network and expressed as anomalies with respect to the 20^th^ century mean [29], [30]. We selected the climate data from the closest grid points to each study site.

### Statistical Analysis

Relationships between climate parameters and each individual and site chronology were analyzed using Pearson's correlation coefficient (CC) over the 1846–1995 period [10]. Climate datasets included monthly data over a 17-month window from May of the year prior to ring formation to September of the current year. The statistical significance of the CCs was tested using a bootstrap procedure with 10000 replications. Each coefficient was considered significant (P<0.05) if the standardized mean value was at least twice the standard deviation of its 10000 replications [31]. The stationarity and consistency of the climate/growth responses were assessed by splitting the original 150-year period in three and performing the same analysis for each 50-year sub-period.

Principal Component Analysis (PCA) [32] of the bootstrap CCs was used to extract common modes of variability in climate/growth responses among the 13 sites. The principal components were calculated on the covariance matrix of variables, as the 34 CCs had previously been standardized and so shared the same unit measures and variance [31]. The number of non-trivial principal components was determined applying a Monte Carlo approach with 999 permutations [33]. As for the climate/growth responses, PCA was performed on the whole 150-year period and on the three 50-year sub-periods.

**Figure 2:**
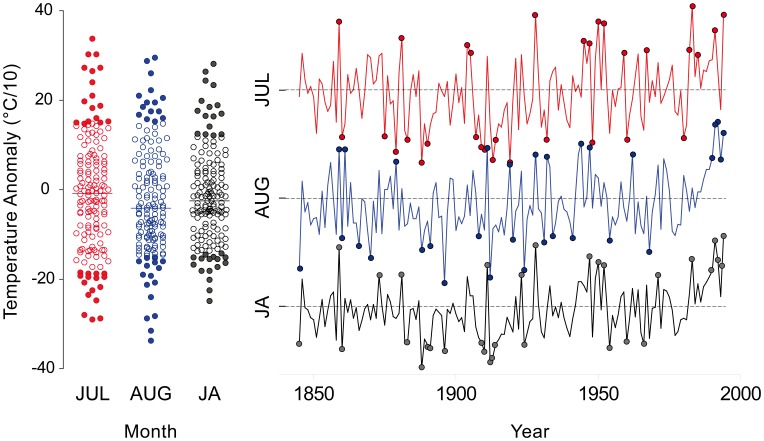
Extreme July and August temperature. Scatter- and time-distribution of the July (J), August (A) and mean JA temperature anomaly for the 1846–1995 period. Full dots represent the extreme values above the 90^th^ and below the 10^th^ percentile respectively. The scale of the Y axis is the same for all the plots.

Finally, to better define the behaviour of the two species, we selected the coldest and warmest years in the 1846–1995 meteorological record following the extreme event definition outlined in the last two IPCC reports (below and above the 10^th^ and 90^th^ percentile, respectively, Fig. 2) [34] for the two most significant and time coherent months in the climate/growth responses (July and August of the previous year, see Results). We then analyzed, the site/species partition among the corresponding calendar years within the indexed tree-ring chronologies by means of fuzzy C-means (FCM) clustering [35]. That is, after having detected the extreme climate events, we extracted the corresponding calendar years from each indexed chronology, creating a new dataset for each site with N = 15–32 values (see Table S1). We then applied the FCM to check for potential site/species partitions among these datasets. FCM is an extension of classic K-means clustering using the concepts of fuzzy logic [36], [37]. In classical set theory, as in the K-means clustering, an object can only be considered a member or non-member of a given set. This membership is usually indicated with a binary variable which takes the value 1 if the object is a member of the set and 0 otherwise. However, in ecology it is not always easy nor desirable to deal with this exclusive partition for several reasons: the high level of disturbance or noise commonly present in ecological data, the common monotonic rather than step-like variability of environmental factors with the associated species' responses and the complex relationships among patterns and processes at ecosystem level. Fuzzy-set theory provides a mathematical approach that is able to better cope with the complexity commonly found in ecological datasets [35] by replacing the binary indicator variable with continuous one, called membership, which can take any real value in the interval [0, 1]. Given this potential, the fuzziness principle is very appealing because it allows a description of some of the uncertainties and ambiguities often found with ecological data [38].

## Results

Table 1 lists the locations and descriptive statistics of the 13 tree-ring site chronologies. All of these statistics, except the rbar and EPS, are significantly higher (P<0.05) for silver fir. Chronologies span from 194 to 360 years in length and have a mean series length ranging from 150 to 238 years. Mean sensitivity (MS) varies from 0.14 to 0.20 while first order serial autocorrelation (AC) ranges from 0.73 to 0.85. Common variance (PC1) and rbar range from 43 to 60 (mean 51) and 0.33 to 0.57 (0.45), respectively, confirming common variability and suggesting a likely significant common climatic forcing. Only three of the 13 site chronologies (two for silver fir and one for spruce), exhibit an EPS value slightly lower than the commonly adopted threshold of 0.85.

**Figure 3:**
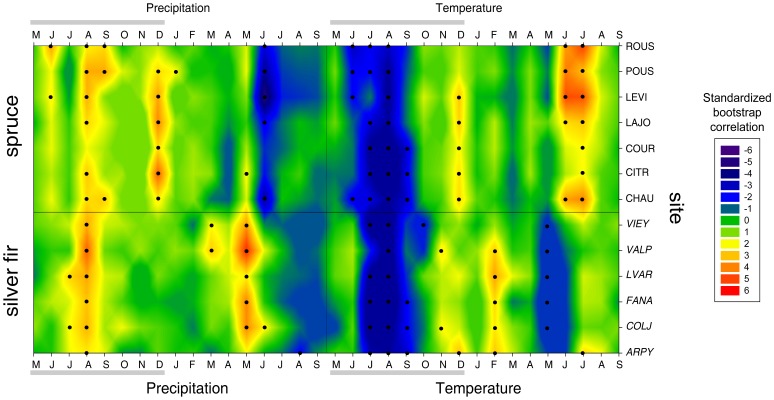
Climate/growth relationships. Correlation functions between site tree-ring indexed chronologies and total monthly precipitation and mean monthly temperatures for the previous (May to December) and current (January to September) growth year. Standardized coefficients were obtained by dividing the mean correlations by their standard deviations after the bootstrap replications and express the significance of monthly parameters. Values above |2| are significant at p<0.05 and are highlighted by black dots.

**Figure 4:**
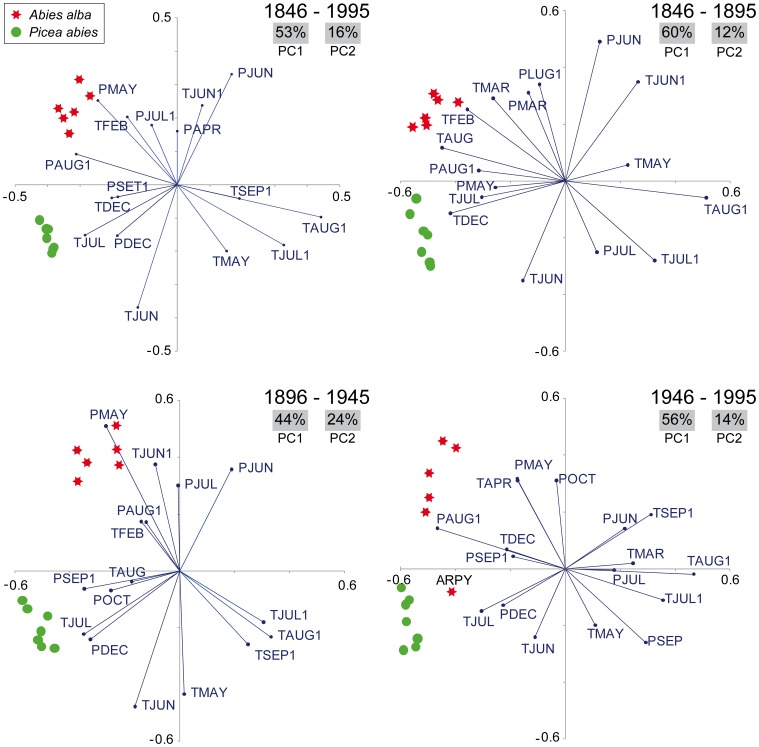
Species-specific and time stability of the climate/growth relationships. Biplots of the principal component analysis (PCA) calculated on the climate/growth responses expressed by the 34 monthly correlation functions coefficients for the entire period (1846–1995) and for the three 50-year sub-periods. Sites belonging to the same species are the same colour. Vectors (lines) represent significant monthly climate factors; the strength of the influence of the climatic parameter is reflected by vector length. Vectors pointing in roughly the direction of a tree-ring chronology indicate a positive correlation, vectors pointing in the opposite direction mean a negative correlation, whereas vectors crossing at right angles correspond to a near zero correlation. The percentage of variance expressed by the first two components is also represented. P or T in the first letter of the vectors' label indicate precipitation or temperature monthly factor, respectively. Vectors' label ending with "1" denotes months in the year prior to the growth year.

Climate/growth responses for all the sites are summarized in Fig. 3. Two distinct features are visible: i) the overall species-specific response with almost all significant correlations clearly separated according to taxon (*e.g*,. the significant positive correlations with June and July temperatures in spruce contrasted with the negative correlations with May temperatures in most of the fir sites) and ii) the most significant correlations for the July and August temperatures of the previous year, which are concurrently the most important common climate forcing between species. Both these emergent features in the climate/growth responses are confirmed by the PCA. Furthermore, they have proven to be stable and consistent throughout the last 150 years after splitting the analyses into the 50-year sub-periods. Fig. 4 shows that the stable partition between the species (just one silver fir site, ARPY, behaves in a different way for the last 50-year sub-period) is consistently related to the second PC axis that explains 12–24% of the total variance. These biplots contain just the significant months, making it possible to appreciate, along with the strength of the climate/growth relationships, the site- and species-specific sensitivity and its course over time. For example, the vector of current year May temperatures for the 1896–1945 and 1946–1995 sub-periods and for the entire 150-year range, points in the opposite direction with respect to the silver fir cluster, highlighting a negative correlation. This fir relationship is at about 90° with respect to the spruce cluster, suggesting no significant association with this species. However, in the 1846–1895 sub-period the same May vector points in the opposite direction for both species, reflecting also for spruce the negative correlation always present for silver fir.

**Figure 5:**
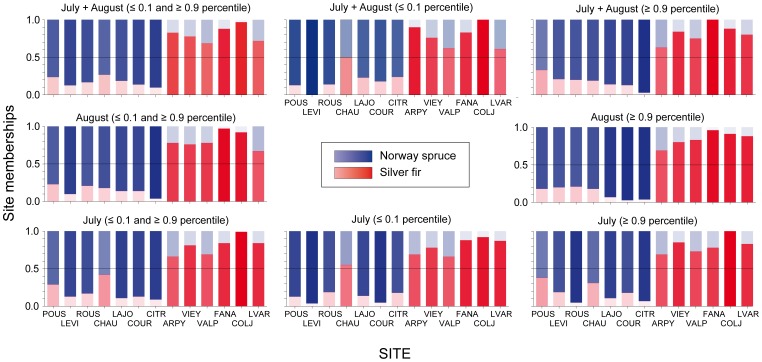
Species-specific sensitivity to extreme climate events. Results of the fuzzy C-means clustering computed with the indexed tree-ring widths occurring in the same calendar years as the extreme climate events (see Fig. 2). All the analyses were performed with the warmest and coldest years concurrently or with just one tail of the distribution at a time. The colour tone within the bars corresponds to the degree of membership of each site/species to the clusters *i.e*., the darker and longer the blue within a bar, the stronger the site's membership to the spruce cluster; the same is true for red with silver fir. A site membership around 0.5 (*e.g*. CHAU with the coldest JA extreme events) means that at this site the species did not show any clear species-specific sensitivity. The legend takes the place of the results for the coldest extremes of August, which are not shown given that no significant partitions were obtained for that month. In the latter case, the responses to those extremes were very similar for both species at all sites.

The above results suggest a clear species-specific separation, yet the two most significant responses (July and August temperatures of the previous year) are similar. The wide range of responses computed for the same months at the individual level are also similar (Fig. S1). Analyses of the extreme years (Fig. 2) gives us a different picture; in this case the FCM clustering (Fig. 5) indicates a clear distinction between the two species. As mentioned, applying FCM clustering the membership of each object (here the site) can be spread between the clusters allowing any intermediate value. This leads to few simple cases with a full membership within one group (for example COLJ for July extreme events) which means that, at this site fir responds in a very different way than spruce, given that its membership is 100% within the fir group. Conversely, there are also a few opposite cases of a split membership shared equally (as for CHAU with the coldest events in July and August), which means that at this site spruce reacts in between the typical spruce and fir responses. Overall, we observed a clear and significant separation between the two taxa (Fig. 5 and Table S1), apart from the single case of the coldest August temperatures of the previous year, where the results did not converge. In the latter case, this means that in most of the sites the two species react in a very similar way. Finally, most of the partitions seem more evident and significant (Table S1) for the warmest events rather than the coldest ones (Fig. 5).

## Discussion

A careful site and tree selection to enhance tree-growth responses to the environmental feature of interest and to maximize age or length of record is the typical mode of sampling in most traditional dendroecological investigations. This approach is appropriate for many types of research in dendroecology [10], [39]. However, the consequences of this approach where the objective is to analyse species responses to climate are a significant overrepresentation of extreme sites and a tendency for different species to converge in their climate/growth relationships within the same site. Here, we show that useful and informative climate information can be retrieved from tree-ring chronologies also on non-extreme sites.

Tree-ring statistics for silver fir indicate a slightly higher year-to-year variability and a greater similarity in the annual growth patterns among sampled trees than spruce. These statistics suggest that fir has a higher sensitivity than Norway spruce to environmental (climate) variability and that overall stand growth for fir is better represented by the mean growth chronologies. This is consistent with many previous studies confirming the better capacity of silver fir to record environmental inputs in its tree-ring sequences compared to the more plastic responses of Norway spruce throughout its broader distribution range [40], [41]. Even so, both species show a similar strength in their sensitivity, likely due to climate variability, as confirmed by the high level of the common variance accounted for by the first principal component (PC1) (44–60%), which highlights that climate can play an important role not only in extreme sites.

The key feature that emerges from our analyses of climate/growth relationships in spruce and fir growing in mesic sites in the Alps is the consistent sensitivity of tree growth to climatic conditions in the previous growing season. This has proven to be a common fingerprint for silver fir throughout its distribution area [42], [43] but represents a novel outcome for Norway spruce: no former studies have detected such a clear previous-year signal for this species [40], [41], [44]. Indeed, while the silver fir behaviour is consistent with its “drought-avoidance” strategy and its lower water-use efficiency [45], spruce is generally recognized for its lower sensitivity to extreme frosts and drought [46]. In our case, this lagged climatic effect could be related to the seasonal dynamics of carbohydrate accumulation and fine root elongation, two essential processes for next season’s bud burst and tree-ring formation for both species. Indeed, as the tree's photosynthetic capacity is highly dependent on bud number and size formed each year, unfavourable conditions during the previous year can affect ring formation by decreasing carbon assimilation in the following growing season [41], [47], [48].

We inspected the tree-ring growth reaction and its partition to extreme climatic events by sharpening the focus on this single seasonal window (July and August temperature). This shows, with mean monthly parameters, both the strongest and most similar relationships between species. The significant and consistent separation (Fig. 5) reveals different behaviour of the two species that is completely disguised when looking only at the mean climate/growth relationships. This outcome further stresses the fundamental and often subtle role of extreme events in enhancing the species-specific responses to climate under comparable growth conditions, and response sensitivity to mean climate variability. Indeed, a wealth of studies have demonstrated that extreme events, although rare by definition, are among the most important factors affecting forest ecosystems, triggering carbon balance anomalies [49] and direct or mediated tree mortality [50], [51]. Furthermore, for the future we might expect an increasing distance between the climate sensitivities of the two species given both their more pronounced separation in the responses during the extreme warm events and the concurrent warming temperature trend recorded in the Alps [22]. The likely increase in the occurrence of those extreme events [52] further suggests a possible shift in the competitive balance of these species, although at present, it is not possible to forecast which species will gain advantage.

The species-specific sensitivity to climate is not just a matter of extreme weather conditions, as it also emerges clearly when observing the whole profile of the monthly responses. Here, based on results from mesic sites, the PCA detected a significant separation between the two conifers. This is in contrast to temperature- or drought-limited sites where different species frequently show a correspondence among the response profiles, especially for the growing season months [15], [53] (but see fig. 2 on Briffa *et al*. [13] for a multi-species northern hemisphere summary). We have shown that the partition between the two species adds up to a fifth of the total common variance (12–24%, the variance accounted for by the second principal component) under the same climate forcing and taking into account the different ecophysiological traits [54], [55]. This is enough to significantly separate the response of the different taxa, though just a minor fraction with respect to PC1 which can be two to five times higher (44–60%).

These results are in line with previous findings of Kunstler *et al*. [8] who trying to disentangle the effect of growth, competition and climatic gradients on trees of different species in the neighbouring French Alps and Jura mountains, highlighted that the decreasing importance of competition with increasing stressful conditions holds mainly for shade-intolerant species. Both spruce and fir, although with slight differences, can be considered shade-tolerant species [56] and this would explain the preeminent role of climatic variability over competition on the tree-growth processes, even though sample sites were far from being typical high elevation stands.

Both of these features, the species-specific responses together with the convergent previous-year climatic sensitivity, were rather stable over time. Several studies in Europe have tested whether climate/growth responses in the two species are stable over time, and the majority reached the conclusion of a temporal instability mainly centred in the last decades [43], [44], [57]. While some of the differences reported in the literature may be related to the specific site ecologies and geographical settings considered in the various studies, the major drivers of these discrepancies are likely differing sampling strategies and, above all, methodological approaches. Indeed, for most northern and central European areas the weakening of the climate signal appears to be predominantly related to local anthropogenic forcing (*e.g.*, SO_2_ emissions from power plants and refineries [57]), whereas typical dieback phenomena in the last decades of the 20^th^ century almost never touched the southern side of the Alps [58]. The methodological approach, with running correlations *vs.* fixed-intervals and PCA, can also lead to different results with their different sensitivities in the trend *vs.* strength detection of the growth/climate relationships.

It is nevertheless important to underline that several limitations of our study may alter its potential to detect the responses to climate of mid-elevation forest ecosystems. First, our investigation covered just two conifers. Although Norway spruce is the most important tree species in Europe and together with silver fir represents a key component of the forest cover in the montane belt of the Alps, further studies with different taxa and regions are needed to generalize our findings. Second, we did not directly consider the effect of topographic differences in temperature and precipitation on local climate. In such a dissected landscape a downscaled climatology would likely permit subtle differences to be unmasked in the growth-climate analyses. Although the study area can be considered rather small and homogeneous with respect to the whole Alpine region, future investigations covering larger regions could be more robust by coupling high-resolution climatologies and digital elevation models [59]. Lastly, we followed the classical dendroecological protocol, selecting sites, sampling trees, and processing data with a likely enhanced climatic signal. This neglects both an even cover of the studied area, immature individuals, and those shaded by competitors or not healthy. This likely provided a biased and artificially inflated picture of the climate sensitivity of these two species, as portrayed in Fig. S1, and suggests that this kind of artefact is not confined to climate-limited environments but is probably a common trait inherent in the classical dendroecological method [15]. On this matter, an unbiased and more robust sampling approach would be desirable. For example, collecting cores to fully assess the range of variation of environmental factors and from most of the age/dimensional classes, would provide a more comprehensive picture of both stand and species sensitivity to those factors.

Our results represent a first step in this direction. Indeed, while still adopting the classical dendroecological sampling protocol, we selected mountain sites at lower elevations. Yet we detected the importance of the species-specific climate sensitivity even within the same regional context. This specificity shows in both the mean and extreme monthly growth responses, with the latter more subtly disentangling the distinct taxon-specific behaviour. The traditional model of tree ecophysiology suggests that species-specific growth responses to climate will begin to converge on more stressful sites (i.e., higher elevation, higher latitude). However, as we demonstrate here, the corollary is that at less stressful sites more pronounced species-specific growth responses occur. A clearer understanding of the nature of these responses along stress gradients will allow for better estimates of long-term growth and the outcomes of competitive interactions in native mixed species forests. Future forest productivity estimates and species reactions to climate change based on tree-ring growth data should take into account a revised and more comprehensive sampling strategy, data from modal sites, and relationships obtained from mesic habitats. These sites and relationships are so far underrepresented in tree-ring studies despite importance of their spatial extent, and contribution to regional biomass in the carbon cycle.

## Supporting Information

Figure S1 Individual responses to climate.
(DOC)

Table S1 Result statistics of the fuzzy C-mean clustering.
(DOC)
