# Supplementary material for: Significant Mean and Extreme Climate Sensitivity of Norway Spruce and Silver Fir at Mid-Elevation Mesic Sites in the Alps
Source: PLoS One. 2012 Nov 29;7(11):e50755. doi: 10.1371/journal.pone.0050755 (PMC3510186; doi:10.1371/journal.pone.0050755)
Supplement: Figure S1 — Individual responses to climate. (DOC) [file pone.0050755.s001.doc]

Figure 1S: Individual responses to climate.


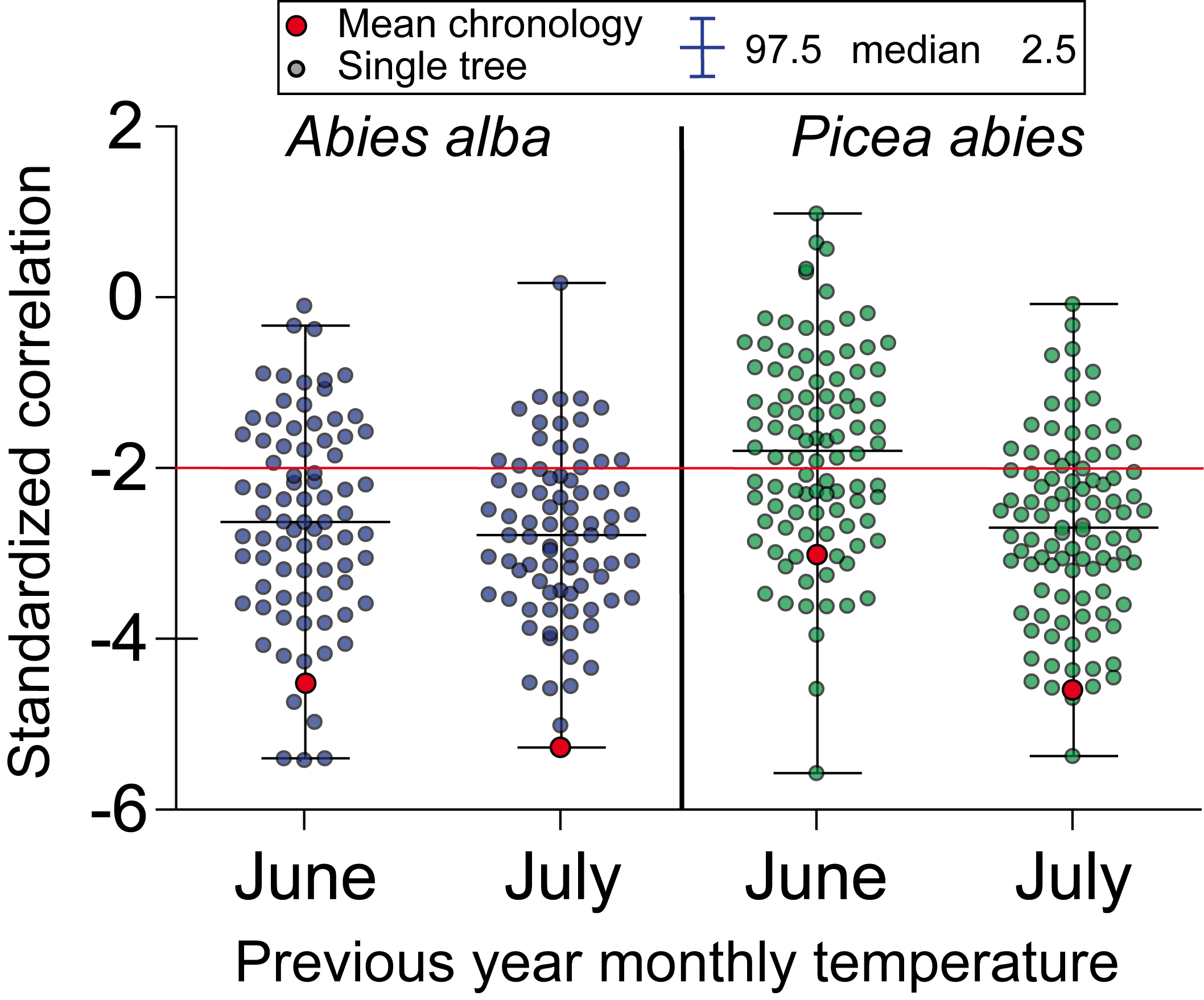


Fig. 1S. Standardized bootstrap correlation coefficients computed for both species between indexed ring-width series and previous year’s June and July for each individual tree for the 1800–1995 period. Whiskers highlight the 2.5– 97.5 percentile range. Red circles represent the mean chronology values. Values below -2 (red horizontal line) are significant at p<0.05.
