# Supplementary material for: Significant Mean and Extreme Climate Sensitivity of Norway Spruce and Silver Fir at Mid-Elevation Mesic Sites in the Alps
Source: PLoS One. 2012 Nov 29;7(11):e50755. doi: 10.1371/journal.pone.0050755 (PMC3510186; doi:10.1371/journal.pone.0050755)
Supplement: Table S1 — Result statistics of the fuzzy C-mean clustering. (DOC) [file pone.0050755.s002.doc]

Table S1. Result statistics of the fuzzy C-mean clustering.

| **Temperature extremes** | **Clusters distance** | **Norm D** | **Group1** | **Group2** | **p-F** | **N** |
| --- | --- | --- | --- | --- | --- | --- |
| ≤ 10^th^ and ≥ 90^th^ Mean Jul-Aug | 0.386 | 0.426 | 6.878 | 6.122 | 10.618 | 30 (29) |
| ≤ 10^th^ Mean Jul-Aug | 0.266 | 0.435 | 6.879 | 6.121 | 10.582 | 15 (14) |
| ≥ 90^th^ Mean Jul-Aug | 0.294 | 0.460 | 6.862 | 6.138 | 11.330 | 15 (15) |
| ≤ 10^th^ and ≥ 90^th^ Aug | 0.418 | 0.473 | 7.064 | 5.936 | 11.607 | 31 (28) |
| ≤ 10^th^ Aug | 0 |  |  |  |  |  |
| ≥ 90^th^ Aug | 0.388 | 0.538 | 7.025 | 5.975 | 14.234 | 16 (11) |
| ≤ 10^th^ and ≥ 90^th^ Jul | 0.423 | 0.424 | 6.849 | 6.151 | 10.659 | 32 (31) |
| ≤ 10^th^ Jul | 0.323 | 0.455 | 6.910 | 6.090 | 11.092 | 16 (15) |
| ≥ 90^th^ Jul | 0.319 | 0.456 | 6.773 | 6.227 | 11.295 | 18 (17) |

Note: Norm D = normalized Dunn Coefficient. A measure of the level of "hardness" in a fuzzy partition; it ranges between 0 (fuzzy) and 1 (hard). Group 1 and Group 2 = number of objects within each cluster. p-F = pseudo F statistic. The higher the value the better the partition [1]. N = samples number, *i.e.* the number of extreme years considered. In parenthesis are the number of calendar years with significant differences between the clusters computed after the partition.

**Reference**

[1] Caliński T, Harabasz J (1974) A dendrite method for cluster analysis. Communications in Statistics-Theory and Methods 3: 1-27.
